# Supplementary material for: Interference length reveals regularity of crossover placement across species
Source: Nat Commun. 2024 Oct 17;15:8973. doi: 10.1038/s41467-024-53054-2 (PMC11487058; doi:10.1038/s41467-024-53054-2)
Supplement: Supplementary file 1 — Supplementary Information [file 41467_2024_53054_MOESM1_ESM.pdf]

Supplementary Information: Interference length reveals regularity of crossover placement across species

Marcel Ernst, Raphael Mercier, and David Zwicker

CONTENTS

1. Traditional quantifications 1
A. Quantification based on a Gamma distribution 1
B. Coefficient of coincidence 1

2. Properties of interference length 2
A. Number of expected pairs 2
B. Effect of random sub-sampling 2
C. Estimation of uncertainty 2
D. Significance testing 4
E. Theoretical distribution without interference 4
F. Maximal-interference model 5
G. Model with regular placement 5
H. Effect of class II crossover 5
I. Calculating interference length for pairs of one class I and one class II CO 6
J. Comparison of quantifications 7

3. Data Handling 7

Supplementary References 12

1. TRADITIONAL QUANTIFICATIONS

A. Quantification based on a Gamma distribution

To determine the shape parameter ν of the gamma distribution for a given set of observed data we simply apply the `python`-function `scipy.stats.gamma.fit` to the data set of all distances between adjacent COs; see Fig. 1B, Supplementary Fig. 1B, and Supplementary Fig. 3A.

B. Coefficient of coincidence

The coefficient of coincidence (CoC) is traditionally defined by chopping up the whole length of the chromosome into intervals: For each pair of intervals A and B, the CoC is defined as the ratio of the observed frequency r_{AB} of double COs to the expected frequency in absence of interference [1–3]. Assuming independence of occurrence in the absence of interference, the latter is given by $r_{\mathrm{A}} \cdot r_{\mathrm{B}}$, where r_A is the frequency of the occurrence of individual COs in interval A, implying [4, 5]

$$\mathrm{CoC}_{\mathrm{AB}} = \frac{r_{\mathrm{AB}}}{r_{\mathrm{A}} r_{\mathrm{B}}} \,. \tag{1}$$

This quantity is 1 if CO positions are independent of each other (e.g., in absence of interference), whereas values smaller than 1 indicate positive interference. To summarize the data for all pairs of intervals, the CoC values are typically plotted against the distance d of the corresponding intervals. Since interval pairs with equal distance typically have similar CoC values, these values are averaged [4, 6]. Furthermore, the interference distance d_{CoC} is defined as the length d where $\mathrm{CoC}(d)$ first exceeds 0.5 [6–9].

One shortcoming of this definition is that the number of intervals between two COs at a given distance depends on their absolute position along the chromosome and not only on their separation. In particular, the distance of a

pair in two adjacent intervals can vary between zero and twice the interval length, i.e., between one interval and the next but one interval, the distances can vary from one to three times the interval length, and so on. This indicates a significant overlap of these intervals.

To avoid this problem, we refine the definition of the CoC by not dividing the entire length of the chromosome into equal intervals and then counting the COs in each interval, but rather by determining the observed and expected distribution of CO distances and then partitioning these distribution into bins. To do so, we determine the actual distances of observed CO pairs and then partition the distance distribution into bins; see the green histogram in Fig. 1A. The expected distance distribution in the absence of interference can be computed by taking all possible distances between all observed CO positions and then partition this distribution by the same set of bins; see the gray histogram in Fig. 1A. The CoC is now simply the ratio of the observed and expected distributions where the observed distribution needs to be scaled with the ratio $\phi = \bar{N}^{\rm pair}_{\rm obs}/\bar{N}^{\rm pair}_{\rm noInt} = 2\bar{N}^{\rm pair}_{\rm obs}/\langle N\rangle^2$ of observed to expected pairs; see definition in section 1A and SI-2A. This implementation of the CoC is used throughout our paper, i.e., in Fig. 1B, Supplementary Fig. 1B and Supplementary Fig. 3B–D. This implementation gives equal weight to all CO pairs and does not depend on the absolute positions of the COs along the chromosome.

2. PROPERTIES OF INTERFERENCE LENGTH

A. Number of expected pairs

We here estimate the expected number of CO pairs for the reference case of absent interference, $\bar{N}^{\rm pair}_{\rm noInt}$, when the average CO count per bivalent is $\langle N\rangle$. In this scenario, the distribution of the number of COs per chromosome follows a Poisson distribution [3], since the COs are assumed to be independent events. Consequently, the probability of finding m COs in a given sample follows a Poisson distribution, $P_{\langle N\rangle}(m) = \frac{e^{-\langle N\rangle}\langle N\rangle^m}{m!}$. Hence,

$$\bar{N}^{\rm pair}_{\rm noInt} = \sum_{m=2}^{\infty} P_{\langle N\rangle}(m)\frac{m(m-1)}{2} = \frac{\langle N\rangle^2}{2} \; . \tag{2}$$

B. Effect of random sub-sampling

We here show that the interference length $L_{\rm int}$ is invariant to random sub-sampling of a certain fraction φ of the COs. First, note that the probability that a given CO *survives* after random sub-sampling is φ, assuming the independent probabilities for all CO. Thus, the distribution of COs along the chromosome remains unchanged, and so does the expected distribution of distances without interference, and thus also $d_{\rm noInt}$. Because the probabilities of individual COs are independent, the joint probability for any pair of COs on the same chromosome is given by φ^2. Consequently, the distribution of distances of all CO pairs remains unchanged and so does the average distance $d_{\rm obs}$. This implies that the ratio ϕ of observed to expected CO pairs also remains unchanged,

$$\phi' = \frac{\bar{N}^{\rm pair'}_{\rm obs}}{\bar{N}^{\rm pair'}_{\rm noInt}} = \frac{2\bar{N}^{\rm pair'}_{\rm obs}}{(\langle N\rangle')^2} = \frac{2\varphi^2\bar{N}^{\rm pair}_{\rm obs}}{(\varphi\langle N\rangle)^2} = \phi \; , \tag{3}$$

where the primed variables correspond to the values after random sub-sampling. Since $d_{\rm noInt}$, $d_{\rm obs}$, and ϕ are invariant under sub-sampling, $L_{\rm int}$ also remains unchanged; see Eq. [3] of the main text.

If we instead considered only the distances between adjacent crossovers, the random sub-sampling would change the distribution of the distances and thus the average $d_{\rm obs}$. This justifies the choice to consider all distances $d_{i,j}$ when determining $L_{\rm int}$.

C. Estimation of uncertainty

To estimate the uncertainty of the quantity $L_{\rm int}$, we determine the standard error of $L_{\rm int}$ using bootstrap sampling. To do this, we first randomly select half of the samples multiple times, calculate $L_{\rm int}$ for each collection, and determine the associated standard error of the mean. Assuming the central limit theorem, the standard error of $L_{\rm int}$ follows by scaling the bootstrapped standard error by $1/\sqrt{2}$ to account for the reduced sample size; see Supplementary Fig. 1A.

In case of positive crossover interference, we expect the uncertainty to scale with $1/\sqrt{n}$. We also expect the uncertainty to be much smaller compared to absent interference since (i) the distribution of the number of crossovers

per chromosome has a smaller variance (see Fig. 2A), and (ii) the distribution of the distances is also narrower; see the latter distribution in Fig. 1A. As an example, this is confirmed by the numerical simulation of the coarsening model [10] for the wild type of *A. thaliana*; see orange data in Supplementary Fig. 1A.

In the following, we analytically estimate the uncertainty of the interference length L_{int} in the absence of interference and with the assumption of a uniform distribution of crossovers along a chromosome of length L. When mean CO count per chromosome is $\langle N \rangle$ and the number of crossovers follows a Poisson distribution $P_{\langle N \rangle}(m)$. For a Poisson distribution $P_{\langle N \rangle}(m)$ the standard deviation of the mean of the number of crossover is given by

$$\bar{\sigma}_{\langle N \rangle} = \frac{\sqrt{\langle N \rangle}}{\sqrt{n}} \,. \tag{4}$$

For m crossovers, the number of pairs is $\frac{m(m-1)}{2}$ and thus the standard deviation of the number of pairs $\bar{N}^{\text{pair}}_{\text{noInt}} = \frac{\langle N \rangle^2}{2}$ (cf. Eq. (2)) is given by

$$\sigma^2_{\bar{N}^{\text{pair}}_{\text{noInt}}} = \sum_{m=0}^{\infty} \left(\frac{m(m-1)}{2} - \frac{\langle N \rangle^2}{2} \right) P_{\langle N \rangle}(m) = \langle N \rangle^2 \left(\langle N \rangle + \frac{1}{2} \right) , \tag{5}$$

and the respective standard error of the mean by

$$\bar{\sigma}_{\bar{N}^{\text{pair}}_{\text{noInt}}} = \frac{\langle N \rangle \sqrt{\langle N \rangle + \frac{1}{2}}}{n} \,. \tag{6}$$

To estimate the standard error of $\phi = 2\bar{N}^{\text{pair}}_{\text{obs}} / \langle N \rangle^2$ we apply error propagation including the covariance term between the number of crossover m and the actual number of pairs p_m

$$\bar{\sigma}^2_{\phi} = \left(\frac{\partial \phi}{\partial \bar{N}^{\text{pair}}_{\text{obs}}} \bar{\sigma}_{\bar{N}^{\text{pair}}_{\text{obs}}} \right)^2 + \left(\frac{\partial \phi}{\partial \langle N \rangle} \bar{\sigma}_{\langle \text{N} \rangle} \right)^2 + 2 \frac{\partial \phi}{\partial \langle N \rangle} \frac{\partial \phi}{\partial \bar{N}^{\text{pair}}_{\text{obs}}} \frac{u(m, p_m)}{n} \tag{7}$$

where $\bar{\sigma}_{\bar{N}^{\text{pair}}_{\text{obs}}} = \bar{\sigma}_{\bar{N}^{\text{pair}}_{\text{noInt}}}$ since we investigate the scenario with absent interference and covariance

$$u(m, p_m) = \sum_{m=0}^{\infty} P_{\langle N \rangle}(m) \, (m - n) \left(p_m - \frac{\langle N \rangle^2}{2} \right) = \langle N \rangle^2 \,, \tag{8}$$

between m and $p_m = \frac{m(m-1)}{2}$. This yields

$$\bar{\sigma}_{\phi} = \frac{\sqrt{2}}{\langle N \rangle} \frac{1}{\sqrt{n}} \,. \tag{9}$$

The distribution of distances of observed crossover pairs d_{obs} corresponds the distribution in absence of interference (cf. (16)). Hence, we get a standard error of the mean of

$$\bar{\sigma}_{d_{\text{noInt}}} = \frac{L}{3\sqrt{2}} \frac{1}{\sqrt{P_{\text{noInt}}}} = \frac{L}{3n} \frac{1}{n} \tag{10a}$$

$$\bar{\sigma}_{d_{\text{obs}}} = \frac{L}{3\sqrt{2}} \frac{1}{\sqrt{P_{\text{obs}}}} = \frac{L}{3n} \frac{1}{\sqrt{n}} \,, \tag{10b}$$

where $P_{\text{noInt}} = \frac{(\langle N \rangle n)^2}{2}$ is the total number of possible shuffled pairs in the data set, and $P_{\text{obs}} = \frac{\langle N \rangle^2}{2} n$ the total number of actually observed pairs. Since $\bar{\sigma}_{d_{\text{noInt}}}$ scales with $\frac{1}{n}$ we neglect this term in the following.

Thus, in the special case of absent interference ($\phi = 0$) and a uniform crossover distribution along the chromosome, the exact analytical estimate of the standard error of L_{int} (cf. Eq. [3] of the main text) is

$$\bar{\sigma}_{L_{\text{int}}} = \sqrt{\left(L - d^{\text{obs}}\right)^2 \bar{\sigma}^2_{\phi} + \phi^2 \bar{\sigma}^2_{d_{\text{obs}}}} = \frac{L}{\langle N \rangle} \frac{1}{\sqrt{n}} \tag{11}$$

with chromosome length L, sample size n, and average CO count $\langle N \rangle$; see blue simulation results and black slope in Supplementary Fig. 1A.

D. Significance testing

The paired t-test allows to test whether the mean of L_{int} of two scenarios (e.g., different mutants) across the same chromosomes in one species deviate significantly from each other. For example, we can test whether L_{int} exhibits sex differences, i.e., whether there is a significant difference between male and female meiosis, or compare two genotypes of the same species. In all cases, we require two scenarios (I and II), between which we want to compare L_{int} of the N^{chr} chromosomes. For each scenario (I/II) and all chromosomes $k = 1, \ldots, N^{\mathrm{chr}}$, we calculate the interference length $L_{\mathrm{int}}^{(k,\mathrm{I/II})}$ together with its standard error $\sigma_{L_{\mathrm{int}}}^{(k,\mathrm{I/II})}$ using bootstrapping. For each scenario (I/II) and all chromosomes $k = 1, \ldots, N^{\mathrm{chr}}$, we calculate the interference length $L_{\mathrm{int}}^{(k,\mathrm{I/II})}$ together with its standard error $\sigma_{L_{\mathrm{int}}}^{(k,\mathrm{I/II})}$ using bootstrapping. The average of the differences of this paired data set then reads

$$\Delta_{L_{\mathrm{int}}} = \frac{1}{N^{\mathrm{chr}}} \sum_{k=1}^{N^{\mathrm{chr}}} \left(L_{\mathrm{int}}^{(k,\mathrm{II})} - L_{\mathrm{int}}^{(k,\mathrm{I})} \right) , \tag{12}$$

and the respective standard error of the mean is

$$\sigma_{\Delta_{L_{\mathrm{int}}}} = \sqrt{ \frac{1}{N^{\mathrm{chr}}} \sum_{k=1}^{N^{\mathrm{chr}}} \left[\left(\sigma_{L_{\mathrm{int}}}^{(k,\mathrm{I})} \right)^2 + \left(\sigma_{L_{\mathrm{int}}}^{(k,\mathrm{II})} \right)^2 \right] } . \tag{13}$$

This results in the t-statistics,

$$T = \frac{\Delta_{L_{\mathrm{int}}}}{\sigma_{\Delta_{L_{\mathrm{int}}}}} . \tag{14}$$

We can use this together with the null hypothesis that the interference length is identical in both scenarios to calculate the corresponding p-value. We use a threshold of 0.05 for rejection of the null hypothesis in our paper.

E. Theoretical distribution without interference

In the following, we determine the distribution of distances between all crossover pairs $d_{i,j}$ for absent crossover interference under the assumption of uniform CO distribution along the chromosome. To do this, we distribute two points independently and uniformly along a straight line with unit length. The distribution of distances between these two points on a non-periodic line is equivalent to the distribution of distances between adjacent points if we place three points $x_1 < x_2 < x_3$ with $x_i \in [0, 1)$ on a line with periodic boundary conditions (a circle) since the end point corresponds to only one other random point. Without loss of generality, let $x_1 = 0$. We then consider the cumulative probability

$$P(x_2 < x) = 1 - P(x_2 \geq x) = 1 - (1 - x)^2 \tag{15}$$

since there are two remaining points in the interval $[x, 1)$. From this follows the probability distribution

$$P(x_2 = x) = \frac{\mathrm{d}P(x_2 < x)}{\mathrm{d}x} = 2(1 - x) . \tag{16}$$

This results in an average distance of

$$\bar{d}_{i,i+1} = \bar{x}_2 = \int_0^1 P(x_2 = x) x \mathrm{d}x = 2 \int_0^1 (x - x^2) \mathrm{d}x = \frac{1}{3} , \tag{17}$$

resulting in $\langle d_{\mathrm{noInt}} \rangle = \frac{1}{3} L$ in the case of a uniform distribution. Consequently, we find $L_{\mathrm{int}} = L - \frac{1}{3} L = \frac{2}{3} L$ in the *complete interference* scenario, where we have exactly one crossover per chromosome, if COs are distributed uniformly along the chromosome.

F. Maximal-interference model

To investigate the maximal value of $L_{\rm int}/L$ for given $\langle N\rangle$, we consider a model where exactly N COs are placed in regular intervals. For $N \geq 2$, we place the two outermost COs exactly at the end of the chromosomes to obtain a normalized distance of $L/(N-1)$ between adjacent COs. In this scenario, we have $\bar{N}^{\rm pair}_{\rm obs} = \frac{N(N-1)}{2}$, $\bar{N}^{\rm pair}_{\rm noInt} = \frac{1}{2}N^2$, and $\phi = \frac{N-1}{N}$. Note that the CO frequency is not uniform for $N \geq 2$, but rather peaked. We have

$$d_{\rm noInt} = \frac{2L}{N(N+1)} \sum_{k=0}^{N-1} (N-k) \frac{k}{N-1} = \frac{L}{3} \,, \tag{18}$$

whereas

$$d_{\rm obs} = \frac{2L}{N(N-1)} \sum_{k=1}^{N-1} (N-k) \frac{k}{N-1} = \frac{N+1}{3(N-1)} L \,, \tag{19}$$

and hence

$$\frac{L_{\rm int}}{L} = \frac{4}{3N}. \tag{20}$$

Note also that $L_{\rm int}/L \leq 1$ since $\phi \geq 0$, $d_{\rm obs} \leq L$ and $d_{\rm noInt} \geq 0$. Hence, we predict $L_{\rm int}/L = 1$ for $N = 1$ (assuming a peaked CO distribution along the chromosome) in the maximal interference model. To extend the model to non-integer values of $\langle N\rangle$, we simply interpolate to obtain a general trend,

$$\frac{L_{\rm int}}{L} = \begin{cases} \frac{4}{3N} & \text{if } N > \frac{3}{4}, \\ 1 & \text{otherwise.} \end{cases} \tag{21}$$

G. Model with regular placement

For the regular-placement model, we place exactly N COs in regular intervals of relative length L/N along the chromosome. Here, we place the first CO uniformly between 0 and L/N, so the overall CO frequency is uniform along the chromosome. In this scenario, the number of observed pairs is $\bar{N}^{\rm pair}_{\rm obs} = \frac{N(N-1)}{2}$ while $\bar{N}^{\rm pair}_{\rm noInt} = \frac{1}{2}\langle N\rangle^2 = \frac{1}{2}N^2$, which yields $\phi = \frac{N-1}{N}$. Because the CO frequency is uniform along the chromosome, the average distance in case of no interference is $d_{\rm noInt} = \frac{1}{3}L$; see SI-2E. The observed average distance between pairs is given by

$$d_{\rm obs} = \frac{L}{\bar{N}^{\rm pair}_{\rm obs}} \sum_{k=1}^{N-1} (N-k) \frac{k}{N} = \frac{N+1}{3N} L \tag{22}$$

since we have $N-k$ pairs of distance $\frac{k}{N}L$. Applying Eq. [3] of the main text , we get

$$\frac{L_{\rm int}}{L} = \frac{1}{N} - \frac{1}{3N^2} \,. \tag{23}$$

To extend the model to non-integer values of $\langle N\rangle$, we interpolate this relation to obtain a general trend. Note that the regular-placement model implies lower $L_{\rm int}/L$ than would be possible for a more peaked CO distribution along the chromosome, so it does not correspond to maximal interference.

H. Effect of class II crossover

A challenge in interpreting crossover interference experimentally is that nearly non-interfering class II COs are indistinguishable in genetics, and invisible with MLH1 from the interfering class I COs.

In the CoC curve, this can be compensated by computing the apparent CoC [11]

$$\mathrm{CoC} = 1 - \varphi^2 (1 - \mathrm{CoC}^{\rm I}) \,, \tag{24}$$

where $\mathrm{CoC}^{\mathrm{I}}$ is the pure coefficient of coincidence of class I crossover as a function of distance, and $\varphi = \frac{\langle N_{\mathrm{I}}\rangle}{\langle N\rangle}$ denotes the fraction of class I COs.

To determine the effect of class II COs on the interference length L_{int}, we assume that the placement of class I and class II crossover is independent, and both classes exhibit the same distribution along the chromosome. Now, the fraction of class II COs is $\varphi^{\mathrm{II}} = 1 - \varphi = \langle N_{\mathrm{II}}\rangle/\langle N\rangle$ ($\varphi, \varphi^{\mathrm{II}} \in [0, 1]$). For simplicity, we assume that class II COs do neither show interference with other class II COs nor with class I COs, implying there are $\langle N_{\mathrm{I}}\rangle\langle N_{\mathrm{II}}\rangle$ mixed pairs. The number of observed pairs, $\bar{N}^{\mathrm{pair}}_{\mathrm{obs}}$, can thus be written as

$$\bar{N}^{\mathrm{pair}}_{\mathrm{obs}} = \bar{N}^{\mathrm{pair}}_{\mathrm{obs,I}} + \frac{\langle N\rangle^2 - \langle N_{\mathrm{I}}\rangle^2}{2} \; . \tag{25}$$

In case of absent interference, we expect $\bar{N}^{\mathrm{pair}}_{\mathrm{noInt}} = \frac{1}{2}N^2$ CO pairs, including $\bar{N}^{\mathrm{pair}}_{\mathrm{noInt,I}} = \frac{1}{2}\langle N_{\mathrm{I}}\rangle^2$ pairs of only class I COs and $\bar{N}^{\mathrm{pair}}_{\mathrm{noInt,II}} = \frac{1}{2}\langle N_{\mathrm{II}}\rangle^2$ pairs of only class II COs. Defining

$$\phi_{\mathrm{I}} = \frac{\bar{N}^{\mathrm{pair}}_{\mathrm{obs,I}}}{\bar{N}^{\mathrm{pair}}_{\mathrm{noInt,I}}} = \frac{2\bar{N}^{\mathrm{pair}}_{\mathrm{obs,I}}}{\langle N_{\mathrm{I}}\rangle^2} \; , \tag{26}$$

we find

$$\phi = \frac{\bar{N}^{\mathrm{pair}}_{\mathrm{obs}}}{\bar{N}^{\mathrm{pair}}_{\mathrm{noInt}}} = \frac{\bar{N}^{\mathrm{pair}}_{\mathrm{obs,I}} + \frac{\langle N\rangle^2 - \langle N_{\mathrm{I}}\rangle^2}{2}}{\frac{\langle N\rangle^2}{2}} = 1 + (\phi_{\mathrm{I}} - 1)\varphi^2 \; . \tag{27}$$

Applying the average distance of class I CO, $\bar{d}_{\mathrm{obs,I}}$,

$$\begin{aligned} \bar{d}_{\mathrm{obs}} &= \frac{\bar{N}^{\mathrm{pair}}_{\mathrm{obs,I}}\,\bar{d}_{\mathrm{obs,I}} + (\bar{N}^{\mathrm{pair}}_{\mathrm{obs}} - \bar{N}^{\mathrm{pair}}_{\mathrm{obs,I}})\,\bar{d}_{\mathrm{noInt}}}{\bar{N}^{\mathrm{pair}}_{\mathrm{obs}}} \\ &= \frac{\phi_{\mathrm{I}}\langle N_{\mathrm{I}}\rangle^2\,\bar{d}_{\mathrm{obs,I}} + (\langle N\rangle^2 - \langle N_{\mathrm{I}}\rangle^2)\bar{d}_{\mathrm{noInt}}}{\langle N\rangle^2 + (\phi_{\mathrm{I}} - 1)\langle N_{\mathrm{I}}\rangle^2} \; . \end{aligned} \tag{28}$$

Using Eq. [3] of the main text , we finally conclude

$$\begin{aligned} L_{\mathrm{int}} &= \phi\bar{d}_{\mathrm{obs}} + (1-\phi)L - \bar{d}_{\mathrm{noInt}} \\ &= \phi\frac{\phi_{\mathrm{I}}\varphi^2\bar{d}_{\mathrm{obs,I}} + (1-\varphi^2)\,\bar{d}_{\mathrm{noInt}}}{1 + (\phi_{\mathrm{I}} - 1)\,\varphi^2} + (1-\phi)L - \bar{d}_{\mathrm{noInt}} \\ &= \varphi^2\left[\phi_{\mathrm{I}}\bar{d}_{\mathrm{obs,I}} + (1-\phi)L - \bar{d}_{\mathrm{noInt}}\right] \\ &= \varphi^2 L^{\mathrm{I}}_{\mathrm{int}} \; , \end{aligned} \tag{29}$$

where $L^{\mathrm{I}}_{\mathrm{int}}$ is the pure interference length measured for only class I COs. Consequently, a higher share of class II COs reduces the apparent interference length L_{int}. However, Eq. (29) allows to estimate the pure interference length of class I COs based on (an estimate of) the fraction of class II crossover.

This analysis assumed that the class II COs do not interfere at all. While an analysis of tomato [12] shows that class II CO indeed not interfere with each other, there might be a small but positive interference between class I and class II CO. This effect reduces the accuracy of the above estimates.

I. Calculating interference length for pairs of one class I and one class II CO

To quantify how class II COs interfere with the positioning of class I COs, we extend the definition of the interference length L_{int} to be able to handle pairs of exactly one class I CO and one class II CO for a chromosome. For that, we need the positions of both types of COs for all samples, denoted by d^{I}_i and d^{II}_i. To obtain L_{int} from Eq. [3] of the main text , we need to compute φ, d_{obs}, and d_{noInt}. To estimate d_{noInt}, we compute the average of all distances between one class I COs and one class II COs over all samples. Similarly, d_{obs} is simply the average of only the observed distances between these mixed pairs, which also gives the number of observed pairs per sample p_{obs}. Finally, we need estimate the ratio of observed to expected pairs, ϕ. The number of expected pairs is now given by $\bar{N}^{\mathrm{pair}}_{\mathrm{noInt}} = \langle N_{\mathrm{I}}\rangle\langle N_{\mathrm{II}}\rangle$ (compare SI-2H), implying

$$\phi = \frac{\bar{N}^{\mathrm{pair}}_{\mathrm{obs}}}{\langle N_{\mathrm{I}}\rangle\langle N_{\mathrm{II}}\rangle} \; . \tag{30}$$

SUPPLEMENTARY TABLE I. **Comparison of various scalar quantifications of CO interference.**

	Interference length L_{int}	Normalized interf. length $L_{\text{int}}^{\text{norm}}$	Interference distance d_{CoC}	Gamma-shape parameter ν
Unit	Length (Mb or µm)	1	Length (Mb or µm)	1
Uncorrelated placement (no interference)	$L_{\text{int}} = 0$	$L_{\text{int}}^{\text{norm}} = 0$	$d_{\text{CoC}} = 0$	$\nu = 1$
Typical values for interference	$L_{\text{int}} \approx (0.2 \ldots 0.6)L$	$L_{\text{int}}^{\text{norm}} \approx 0.6 \ldots 0.8$	$d_{\text{CoC}} \approx (0.2 \ldots 0.6)L$	$\nu \approx 3 \ldots 20$ [13]
One CO per chromosome	$L_{\text{int}} \approx \frac{2}{3}L$	$L_{\text{int}}^{\text{norm}} \approx \frac{2}{3}$	$d_{\text{CoC}} = L$	undefined
One CO at each chromosome end	$L_{\text{int}} \approx \frac{2}{3}L$	$L_{\text{int}}^{\text{norm}} \approx \frac{2}{3}$	$d_{\text{CoC}} = L$	$\nu \to \infty$
Regular-placement model	$L_{\text{int}} = L[N^{-1} - \frac{N^{-2}}{3}]$	$L_{\text{int}}^{\text{norm}} = 1 - \frac{1}{3}N^{-1}$	unclear	$\nu \to \infty$
Random sub-sampling	Invariant	Invariant	Invariant	Sensitive to random sub-sampling
Data sensitivity	Incorporates all data	Incorporates all data	Only sensitive to data close to transition point at CoC = 0.5	Only uses data from chromosomes with at least two COs
Binning necessary	No	No	Yes	No
Assumption for CO distribution	None	None	None	Assumes uniform distribution along SC

We can thus apply Eq. [3] of the main text to compute the interference length for the mixed pairs of one class I CO and one class II CO.

J. Comparison of quantifications

Supplementary Table I offers a direct comparison of some properties of the various scalar quantifications of CO interference, in particular the interference length L_{int}, the interference distance d_{Coc}, and the gamma shape parameter ν.

3. DATA HANDLING

To investigate the effect of crossover interference in detail, we used empirical data sets for multiple species and genotypes. For various genotypes of *A. thaliana*, we used cytological data [14, 15] and genetic data [10, 16, 17]. The cytological data [14] was filtered by only using the data where the column `Stage` was in the `late` phase of meiosis and the `quality` was 1.0. In the cytological data for the *zyp1* mutant [15], most CO position were measured twice, so we averaged those measurements. The measured path lengths of the bivalents were used to assign a SC length since the axis length is not changed significantly in the *zyp1* mutant [10]. The genetic data for *A. thaliana* is provided by [10, 16]. In the hybrid Col/*Ler* wild-type line from [16] (`wt1`), we deleted the sample `Plate2_C4` because of probable mis-genotyping. In the hybrid Col/*Ler* wild-type line from [10] (`wt2`), we excluded the data from chromosome number 4 because of a potential inversion in the *Ler* line. The HEI10^{oe}, *zyp1*, and *zyp1*HEI10^{oe} data is taken from [10]. The HEI10^{het} data is provided by the authors of [17]. The genetic background of the mutants is not identical for the cytological and genetic data, but rather represent similar levels of HEI10 over- and underexpression; details are provided in the respective publications [10, 14–17]; compare Fig. 4 in the main text.

Additionally, we used data for *A. arenosa* [18] (cytological), *C. elegans* [19] (cytological), human [20] (cytological) for both male and female, human [21] (genetic) and respective chromosome lengths from [22], maize [23] (cytological, data provided in [24]), mouse [25] (cytological, data provided in [26]), tomato [12] (cytological, data provided in [27]) and for *S. cerevisiae* [28] (genetic data from full tetrads). For *C. elegans*, the wild-type data is given for two genotypes, with *ie29* having an HA epitope tag at the SC [19]. Since the different chromosomes in *C. elegans* are of similar length,

it is not possible to separate individual chromosomes, and so the data were grouped together. For the cytological data of *A. thaliana* [14, 15] and *A. arenosa* [18] we assigned the individual paths to chromosome number by ranking the measured path lengths. We assume the starting position of the respective chromosomes as given in the data set. If the starting positions of the paths are unclear, an unbiased comparison could be created by adding the mirrored data to the dataset.

For most species, the data of CO positions was provided as relative position along the respective chromosome in the interval $[0, 1]$ together with the average length of the chromosome or SC over all samples. However, for the cytological data for *A. thaliana* [14, 15] and the data for *C. elegans* [19], the CO positions were given as absolute positions (in µm) together with the SC length for each individual sample. To make these different data comparable, we also calculated the relative CO positions along each path and the average SC lengths in these cases.

To determine the average CO count $\langle N \rangle$ per *bivalent* when analyzing genetic data (human [21] and *A. thaliana* [10, 16, 17]), we double the observed value to compensate for sub-sampling of COs. However, for genetic data from *Saccharomyces cerevisiae*/yeast [28] this is not necessary since the data are based on genetic analysis of full tetrads.

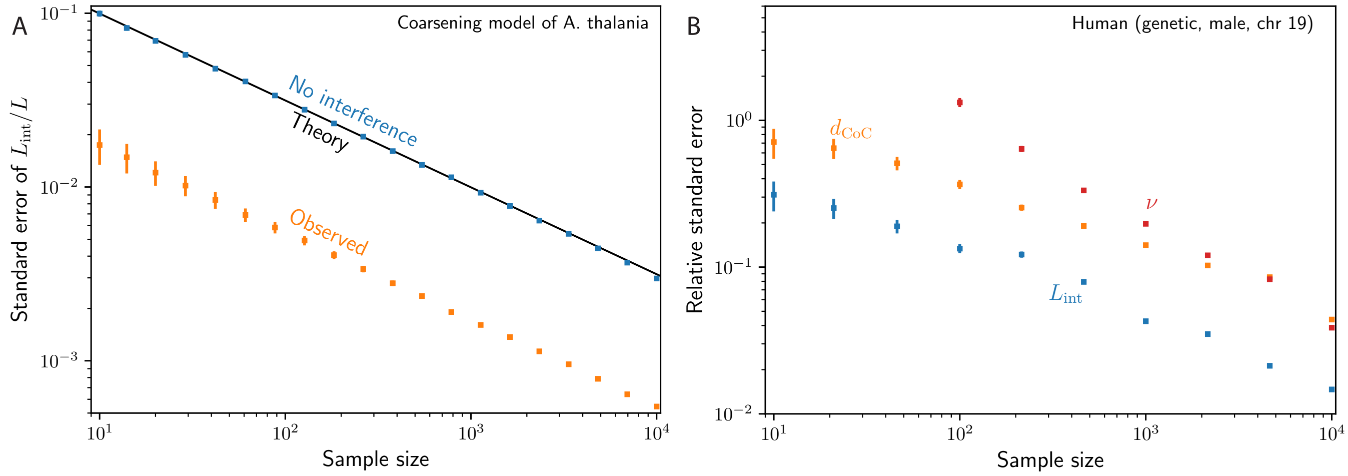

SUPPLEMENTARY FIGURE 1. **Uncertainty of interference length L_{int} is smaller compared with traditional quantifications.** (A) Uncertainty (standard deviation of the mean) based on bootstrap sampling for the normalized interference length (L_{int}/L) as a function of sample size in (i) the case of absent interference by simply assuming uniform distribution of crossovers along the chromosome (blue) and (ii) the case of crossover interference by applying the coarsening model in [10] (50000 overall samples) for a chromosome of length 50 µm (orange). In both cases we have an average of $N \approx 3.2$ crossovers per chromosome. We also show the theoretical uncertainty for absent interference (black). (B) Uncertainty based on bootstrap sampling for human genetic data (31228 overall samples) for chromosome 19 which has an average CO count of $\langle N \rangle \approx 1$. Shown is the standard deviation as a function of sample size of the (i) normalized interference length L_{int}/L (blue) (ii) normalized interference distance d_{CoC}/L (orange) using 15 intervals to compute the CoC curves (iii) gamma shape parameter ν normalized with the actual ν based on the full data set (red). (A–B) Error bars indicate standard error of the mean.

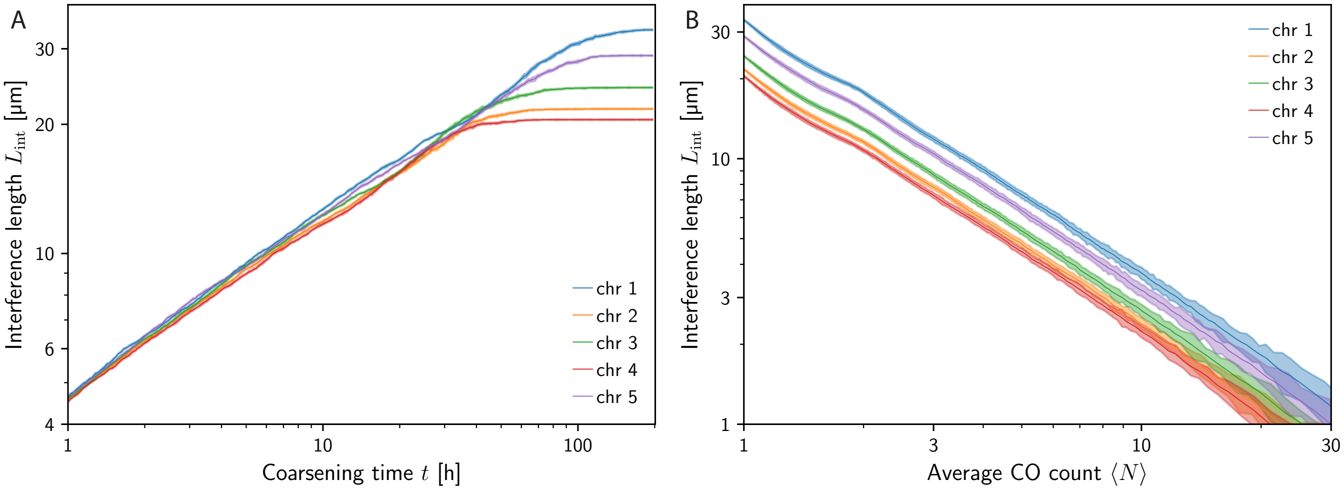

SUPPLEMENTARY FIGURE 2. **Coarsening model predicts linear growth of the interference length $L_{\rm int}$ with similar values across chromosomes and convergence to $\frac{2}{3}L$ for the one CO limit.** (A) Numerical data of the time evolution of $L_{\rm int}$ in the coarsening model for each individual chromosome of *A. thaliana* [10, 16] (wild type). (B) Interference length as a function of the average CO count per chromosome $\langle N \rangle$ for each individual chromosome. (A–B) Shaded area indicates standard error of the mean.

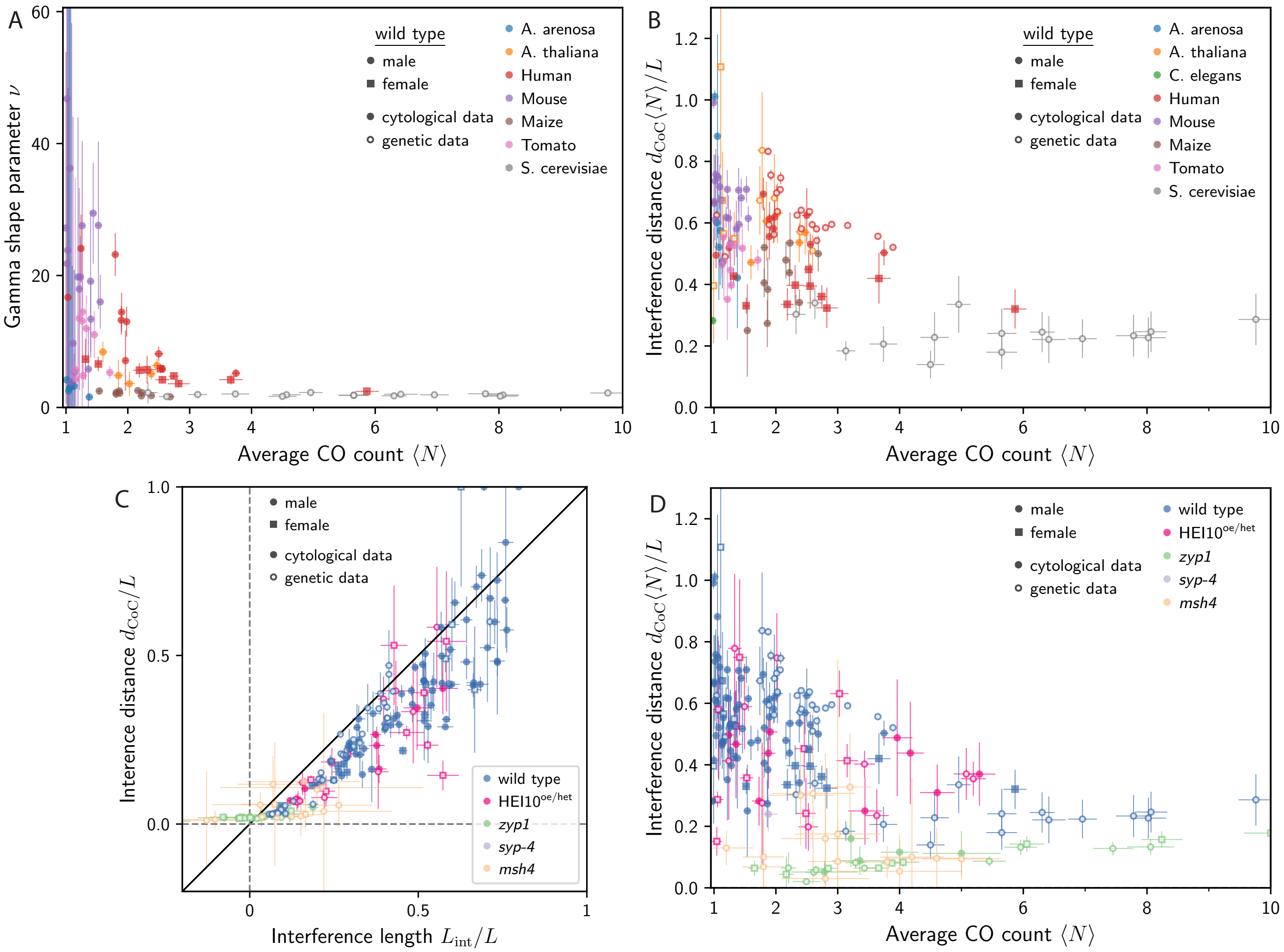

SUPPLEMENTARY FIGURE 3. **Traditional measures do not show the similarity observed for interference length.** (A) Gamma shape parameter ν as a function of the average CO count $\langle N \rangle$ in logarithmic scales for wild-type data for the cytological data as in Fig. 6A and full-tetrad genetic data of budding yeast. The data of *C. elegans* is dropped because it has not enough CO pairs, as well as the genetic data of *A. thaliana* and human because the shape parameter ν is not invariant to random sub-sampling (cf. SI-1A). In particular, for small $\langle N \rangle$ the uncertainties are huge due to small sample sizes of observed adjacent COs. (B) Interference distance d_{CoC} as a function of average CO count $\langle N \rangle$ (using 15 intervals to compute the CoC curves) for the wild-type data for both cytological and genetic data as in Fig. 6A. (C) Correlation of interference distance d_{CoC}/L and interference length L_{int}/L: Comparison of interference distance d_{CoC}/L and interference length L_{int}/L normalized with respective chromosome length for the data points shown in Fig. 6B. (D) Interference distance d_{CoC} as a function of average CO count $\langle N \rangle$ (using 15 intervals to compute the CoC curves) for the mutant behaviour data for both cytological and genetic data as in Fig. 6B. (A–D) Error bars indicate standard error of the mean. Details of data handling in SI-3.

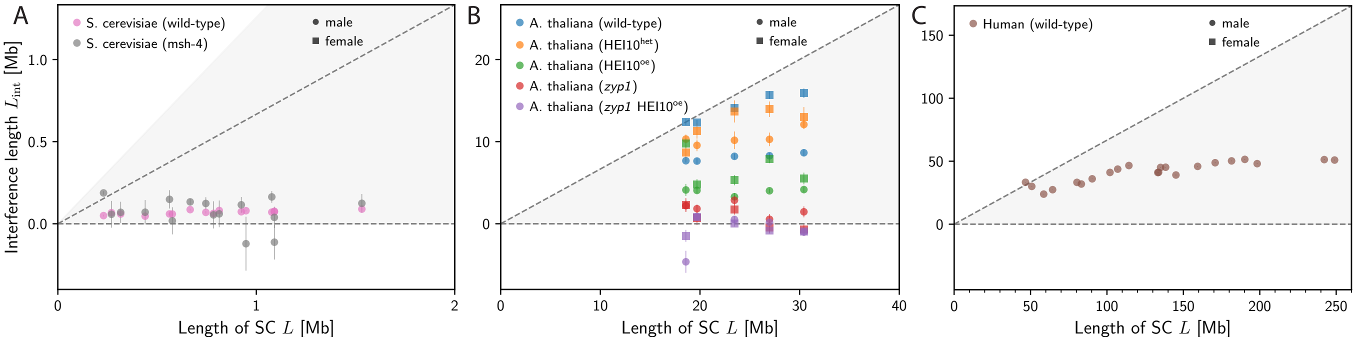

SUPPLEMENTARY FIGURE 4. **Interference length allows for simple comparison across species and genotypes.** (A–C) Interference length $L_{\rm int}$ as a function of chromosome length L for genetic data of various wild-type and mutant data of *A. thaliana* [10, 16, 17], human [21] and *S. cerevisiae* [28]. Error bars indicate standard error of the mean. Details of data handling in SI-3.

[1] A. H. Sturtevant, The behavior of the chromosomes as studied through linkage, Zeitschrift für induktive Abstammungs-und Vererbungslehre **13**, 234 (1915).

[2] H. J. Muller, The mechanism of crossing-over, The American Naturalist **50**, 193 (1916).

[3] J. B. Haldane, The combination of linkage values and the calculation of distances between the loci of linked factors, J Genet **8**, 299 (1919).

[4] S. Wang, Y. Shang, Y. Liu, B. Zhai, X. Yang, and L. Zhang, Crossover patterns under meiotic chromosome program, Asian Journal of Andrology **23**, 562 (2021).

[5] Y.-C. Chuang and G. R. Smith, Chapter nine - meiotic crossover interference: Methods of analysis and mechanisms of action, in *Current Topics in Developmental Biology*, Vol. 151, edited by F. Cole (Academic Press, 2023) pp. 217–244.

[6] L. Zhang, S. Wang, S. Yin, S. Hong, K. P. Kim, and N. Kleckner, Topoisomerase ii mediates meiotic crossover interference, Nature **511**, 551 (2014).

[7] C. Girard, The regulation of meiotic crossovers distribution: a coarse solution to a century-old mystery?, None (2023).

[8] D. Zickler and N. Kleckner, A few of our favorite things: Pairing, the bouquet, crossover interference and evolution of meiosis, Seminars in Cell & Developmental Biology **54**, 135 (2016).

[9] S. Wang, D. Zickler, N. Kleckner, and L. Zhang, Meiotic crossover patterns: obligatory crossover, interference and homeostasis in a single process, Cell cycle (Georgetown, Tex.) **14**, 305 (2015).

[10] S. Durand, Q. Lian, J. Jing, M. Ernst, M. Grelon, D. Zwicker, and R. Mercier, Joint control of meiotic crossover patterning by the synaptonemal complex and hei10 dosage, Nature Communications **13**, 5999 (2022).

[11] F. Stahl, Defining and detecting crossover-interference mutants in yeast, PloS one **7**, e38476 (2012).

[12] L. K. Anderson, L. D. Lohmiller, X. Tang, D. B. Hammond, L. Javernick, L. Shearer, S. Basu-Roy, O. C. Martin, and M. Falque, Combined fluorescent and electron microscopic imaging unveils the specific properties of two classes of meiotic crossovers, Proceedings of the National Academy of Sciences **111**, 13415 (2014).

[13] S. P. Otto and B. A. Payseur, Crossover interference: Shedding light on the evolution of recombination, Annual Review of Genetics **53**, 19 (2019).

[14] C. Morgan, J. A. Fozard, M. Hartley, I. R. Henderson, K. Bomblies, and M. Howard, Diffusion-mediated hei10 coarsening can explain meiotic crossover positioning in arabidopsis, Nature Communications **12**, 4674 (2021).

[15] J. A. Fozard, C. Morgan, and M. Howard, The synaptonemal complex controls cis- versus trans-interference in coarsening-based meiotic crossover patterning, bioRxiv , 2022.04.11.487855 (2022).

[16] D. K. Singh, Q. Lian, S. Durand, J. B. Fernandes, A. Chambon, A. Hurel, B. Walkemeier, V. Solier, R. Kumar, and R. Mercier, Heip1 is required for efficient meiotic crossover implementation and is conserved from plants to humans, Proceedings of the National Academy of Sciences **120**, e2221746120 (2023).

[17] S. Durand, Q. Lian, V. Solier, J. B. Fernandes, and R. Mercier, Mutlgamma enforces meiotic crossovers in arabidopsis thaliana, bioRxiv , 2024.09.18.613675 (2024).

[18] C. Morgan, M. A. White, F. C. H. Franklin, D. Zickler, N. Kleckner, and K. Bomblies, Evolution of crossover interference enables stable autopolyploidy by ensuring pairwise partner connections in arabidopsis arenosa, Current Biology **31**, 4713 (2021).

[19] S. Köhler, M. Wojcik, K. Xu, and A. F. Dernburg, Dynamic molecular architecture of the synaptonemal complex, BioRxiv 10.1101/2020.02.16.947804 (2020).

[20] S. Wang, T. Hassold, P. Hunt, M. A. White, D. Zickler, N. Kleckner, and L. Zhang, Inefficient crossover maturation underlies elevated aneuploidy in human female meiosis, Cell **168**, 977 (2017).

[21] A. D. Bell, C. J. Mello, J. Nemesh, S. A. Brumbaugh, A. Wysoker, and S. A. McCarroll, Insights into variation in meiosis from 31,228 human sperm genomes, Nature **583**, 259 (2020).

[22] V. A. Schneider, T. Graves-Lindsay, K. Howe, N. Bouk, H.-C. Chen, P. A. Kitts, T. D. Murphy, K. D. Pruitt, F. Thibaud-Nissen, D. Albracht, *et al.*, Evaluation of grch38 and de novo haploid genome assemblies demonstrates the enduring quality of the reference assembly, Genome research **27**, 849 (2017).

[23] L. K. Anderson, G. G. Doyle, B. Brigham, J. Carter, K. D. Hooker, A. Lai, M. Rice, and S. M. Stack, High-resolution crossover maps for each bivalent of zea mays using recombination nodules, Genetics **165**, 849 (2003).

[24] L. K. Anderson, G. G. Doyle, B. Brigham, J. Carter, K. D. Hooker, A. Lai, M. Rice, and S. M. Stack, Data from: High-resolution crossover maps for each bivalent of zea mays using recombination nodules [dataset]., Dryad doi.org/10.5061/dryad.6hdr7sr69 (2023).

[25] L. Froenicke, L. K. Anderson, J. Wienberg, and T. Ashley, Male mouse recombination maps for each autosome identified by chromosome painting, The American Journal of Human Genetics **71**, 1353 (2002).

[26] L. Froenicke, L. K. Anderson, J. Wienberg, and T. Ashley, Data from: Male mouse recombination maps for each autosome identified by chromosome painting [dataset], Dryad doi.org/10.5061/dryad.gb5mkkwx5 (2024).

[27] L. K. Anderson, L. D. Lohmiller, X. Tang, D. B. Hammond, L. Javernick, L. Shearer, S. Basu-Roy, O. C. Martin, and M. Falque, Data from: Combined fluorescent and electron microscopic imaging unveils the specific properties of two classes of meiotic crossovers [dataset], Dryad doi.org/10.5061/dryad.0vt4b8h5m (2023).

[28] E. Mancera, R. Bourgon, A. Brozzi, W. Huber, and L. M. Steinmetz, High-resolution mapping of meiotic crossovers and non-crossovers in yeast, Nature **454**, 479 (2008).
